# Processing Homophones Interactively: Evidence from eye-movement data

**DOI:** 10.1038/s41598-018-27768-5

**Published:** 2018-06-28

**Authors:** Michael C. W. Yip, Mingjun Zhai

**Affiliations:** 1 Department of Psychology, The Education University of Hong Kong, Hong Kong, China; 2 Department of Psychology, Rice University, Texas, USA

## Abstract

The question of how to process an ambiguous word in context has been long-studied in psycholinguistics and the present study examined this question further by investigating the spoken word recognition processes of Cantonese homophones (a common type of ambiguous word) in context. Sixty native Cantonese listeners were recruited to participate in an eye-tracking experiment. Listeners were instructed to listen carefully to a sentence ending with a Cantonese homophone and then look at different visual probes (either Chinese characters or line-drawing pictures) presented on the computer screen simultaneously. Two findings were observed. First, the results revealed that sentence context exerted an early effect on homophone processes. Second, visual probes that serve as phonological competitors only had a weak effect on the spoken word recognition processes. Consistent with previous studies, the patterns of eye-movement results appeared to support an interactive processing approach in homophone recognition.

## Introduction

How context affects spoken word recognition processes during lexical access has been researched intensively over the past 30 years. When a listener hears an ambiguous word, with multiple meanings, in a sentence, do all the meanings associated with that ambiguous word activate in the listener’s mind at the very beginning stage of the lexical access, or does the preceding sentence context facilitate the suppression of irrelevant meanings at a later stage? This is one of the fundamental empirical questions in the study of language processing, i.e. lexical ambiguity resolution^1^. Nonetheless, there is still no definitive answer after decades of psycholinguistic research.

There were two contrasting hypotheses suggested by the past research in western studies^2–10^. The first one is the exhaustive access hypothesis. This proposes that all meanings relevant to an ambiguous word will be accessed automatically and immediately after the occurrence of the word, and that the context can only assist with the selection of the most appropriate meaning at the post-access stage. Onifer and Swinney^11^ conducted a cross-modal priming study to demonstrate these effects. In the experiment, two different types of sentence context were presented to participants in audio-mode^12^: the sentence context was biased to the dominant meaning of the ambiguous word, and^13^ the sentence context was biased to the subordinate meaning of the ambiguous word.

For example,

1. The businessman took the checks and receipts to the first national city bank on his way to the board meeting.
2. The rudderless boat reached the edge of the water and ran onto the gently sloped bank without as much as scratching the paint on the hull.

A visual probe was presented immediately after the occurrence of the ambiguous word on the screen. This was related to one of the meanings (dominant and subordinate) of the ambiguous word or to an unrelated control, such as MONEY (Dom) – FIELD (control), RIVER (Sub) – BASIC (control). The results showed that the participants’ response times to visual probes related to both dominant and subordinate meanings of the ambiguous word were facilitated equally after the occurrence of the ambiguous word in the sentence. Therefore, the researchers, based on these findings, argue for the exhaustive access hypothesis. This hypothesis implies that language processing should function as a modular mechanism and bottom-up process in which non-lexical or contextual information cannot penetrate lexical access^14^. Lexical access should operate according to the autonomous principle.

The alternative view is the context-dependency hypothesis. This hypothesis argues that only the contextually appropriate meaning of an ambiguous word will be accessed at an early stage when the sentence context has a strong semantic bias towards the contextually appropriate meaning. Simpson^15^ employed similar research procedures to replicate the previous experiment conducted by Onifer and Swinney, and observed a different pattern of results. In his study, Simpson^15^ observed a faster response time to the probes related to the dominant meaning of the ambiguous word than those related to the subordinate meaning of the ambiguous word and the unrelated control word after the occurrence of the sentence-ended ambiguous word in the semantically-constraint sentence condition. Glucksberg, Kreuz and Rho^3^ offered further evidence supporting the context-dependency hypothesis from the results of a series of cross-modal experiments. They observed that context could constrain the initial activation to different meanings of an ambiguous word in different magnitudes. Additionally, Tabossi^10^ presented similar findings to support the early context effects on selecting the contextually appropriate meaning of an ambiguous word in Italian. Taken altogether on those findings, the researchers argued for the context-dependency hypothesis in lexical ambiguity resolution. This hypothesis assumes that language processing should function as an interactive-activation mechanism, in which information can flow bi-directionally and concurrently, i.e. both bottom-up and top-down processing exist in the system. More importantly, lexical access and contextual information can influence each other mutually^16^. Therefore, lexical access should operate interactively because context robustly limits lexical access^17^.

These hypotheses have been examined mostly in English and several Indo-European languages. Essentially, Chinese language offers a few unique psycholinguistic properties in different linguistic dimensions, such as phonological, lexical, and syntactic structures^18^ to test these hypotheses. The properties of Chinese language (lexical tones, morphemic mono-syllabicity and the word compounding nature) pose a very good challenge to verify these hypotheses in sentence processing from a different perspective^19^. For example, Chinese is a tone language in which tonal information can differentiate different meanings associated with the same syllable. However, tonal information alone cannot completely eliminate lexical ambiguities associated with homophones because Chinese has a huge number of homophones on a lexical-morphemic level even with the tonal distinctions. Based on the information of the *Modern Chinese Dictionary*^20^, 80% of monosyllables in Chinese are ambiguous because of their various possible meanings, and 55% of monosyllables have at least five meanings. For example, in Cantonese (one of the major dialects in the Chinese language), the monosyllabic word /si^1^/has up to 14 individual meanings (and has at least seven commonly-used meanings, such as corpse, lion, poem, private, silk, teacher, think); this number would increase to 37 if the identical monosyllables with different tones were considered as homophones^21^. This number excludes the word compounding nature of Chinese word^22^. According to the context-dependency hypothesis, upon hearing /si^1^/ in a sentence, native Cantonese listeners would not activate all 14 (or seven) possible meanings of this single word. Only the contextually appropriate meaning will be activated when listeners hear this homophone, due to the robust and early context effects.

There has been a scarcity of research on Chinese lexical ambiguity, but Li and Yip^23,24^ examined the time course of context effects on Cantonese listeners’ lexical access of homophones processing in a pioneering study, employing a cross-modal priming task and a gating experiment. The results from the cross-modal priming task showed that context effects appeared immediately after the occurrence of the Cantonese homophone, and the gating experiment further showed that participants could identify the appropriate meaning that fit the sentence context with less than 50% of the acoustic information of the homophone. Converging evidence was also observed in another series of experimental studies using disyllabic homophones in Mandarin^25^. The results from these experiments indicated that native Chinese listeners (including Cantonese and Mandarin) were cognitively sensitive to the contextually biased meaning at an early stage during lexical access, probably within the acoustic boundary of the ambiguous word, a much earlier context effect compared with the previous studies in English^11^. Nonetheless, other researchers observed some contrasting evidence to support the exhaustive access of meaning in Chinese homophone processing^12,13,26^. However, Mandarin Chinese has been used as the target language in these studies, and the research design and task demands were a bit of different from those used by Li and Yip, even though they all drew conclusions about behavioral data. To examine the issue further, the present study aimed to use the visual world paradigm to investigate lexical ambiguity resolution in Chinese. Many recent experimental studies of different languages have found the visual world paradigm to be an effective and useful way to track the ongoing cognitive processes of spoken word recognition and sentence comprehension^27–31^. The eye-tracking technique allows researchers to monitor and measure participants’ eye-movement patterns precisely during their actual viewing. This technique provides a clear temporal resolution of several distinct cognitive processes in understanding online language situations that closely resemble natural reading and listening scenarios, that is the linking hypothesis^30–32^. The present study carefully adopted this technique^29^ to examine the context effects issue again in order to obtain solid and convergent evidence (along with other previous studies using different behavioral measurements) to understand the whole and comprehensive picture of homophone processing in context. Basing on a large-scale related research project, Yip and Zhai^33^ used a similar approach to study similar issue in lexical disambiguation in Mandarin Chinese and they proved successfully the eye-tracking technique could effectively tap the dynamics of online spoken word processing and sentence processing as well as to avoid the potential confounding pointed out by previous research^6,19^. Using this paradigm, we could analyze the different eye-movement patterns for each visual probe along the time frame of the spoken sentence; the time-course of the spoken word recognition processes and their relationship to the context effects could thus be revealed. For example, it was predicted that an early (i.e. before the acoustic offset point or earlier) priming effect (reflected by higher fixation probability to a specific type of visual probes, such as the contextually-related visual probes) would predict strong context effects in resolving lexical ambiguities at an early stage during lexical access, thereby supporting the context-dependency hypothesis. It was also predicted that a different pattern of results would support the exhaustive access approach if the priming effects occurred at a later post-access stage (i.e. after the acoustic offset point).

## Experiment

### Method

#### Participants

Sixty native Cantonese speakers were recruited to participate in this experiment. They were all university students with normal or corrected-to-normal vision. They were invited to complete a Language History Questionnaire, LHQ2.0^34^ to record the demographical information and language experience. None reported any visual, speech or hearing deficits. They were paid HK$50 for their participation as an incentive. Written informed consent was obtained from each participant and they were all informed verbally about the procedure. The study was approved by the HREC of the Education University of Hong Kong and all methods and procedures were carried out according to the guidelines and regulations approved by the University.

### Materials

Thirty spoken Cantonese homophones were chosen as the testing materials. Each had two different meanings with the same tone (the homophone density was controlled and all items were nouns). Each homophone was placed at the end of three different sentences with the preceding context either semantically biased to the dominant or the subordinate meaning or with both meanings of the homophone ambiguously^23,24,33^. In addition, another separate group of native Cantonese speakers (n = 20) was invited complete a cloze test to evaluate the constraint effect of the preceding sentence context on the target homophone. They were given the sixty test sentences with the preceding biasing context (excluding the 30 ambiguous test sentences) but without the homophone, and then asked to fill in a Cantonese word, which would naturally complete the sentence. All of their proposed words were scored on a 4-point scale, based on the one proposed by Marslen-Wilson and Welsh:^35^ 1 point was given for a word identical to the test word, 2 points for a synonym, 3 for a related word, and 4 for an unrelated word. The responses were pooled across the twenty raters, and the mean rating was 1.6 (SD = 0.43). This score was achieved at the high constraint condition set by Marslen-Wilson and Welsh^35^. The sentence lengths were 13 to 15 characters.

An example of a Cantonese homophone and the corresponding sentences are shown below:

Homophone:  [cheung1]

1. Biased toward the dominant meaning:

2. Biased toward the subordinate meaning:

3. Ambiguous:

Quartets of visual probes were selected for the 30 experimental trials. Each set of probes consisted of line-drawn pictures or words^12^ related to the dominant meaning of the homophone^13^, related to the subordinate meaning of the homophone^32^, phonologically similar to the homophone, or^21^ unrelated to the homophone. We used both line-drawn pictures and printed words as visual probes in the present study because they can respectively capture the processing of phonological, semantics and conceptual representations of the stimuli during different stage of lexical access^28,36,37^. The frequency information of the words was based on the work of Ho and Jiang^38^. All the visual probes were selected carefully through a semantic relatedness norm study, with another group of native Cantonese speakers (*n* = 100), conducted via the Internet. None of this group had participated in the eye-tracking experiment. In the semantic relatedness norm study, the participants were asked to think immediately of three two-character nouns that were related closely to the meaning of each homophone. The most frequent words proposed by the group were used as the semantically related visual probes for each meaning of the spoken homophone. The unrelated probes (unrelated to other visual probes semantically or phonologically) were selected randomly from the same pool provided by these participants. The phonologically similar probes shared the rime (both vowel and tone) with the target homophone. All the visual probes in each experimental condition were matched in terms of the number of character strokes and word frequency.

**Figure 1:**
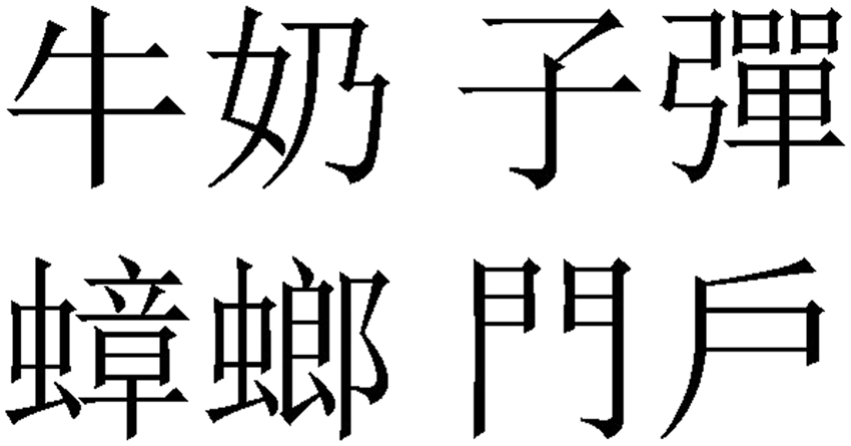
Word-viewing version.

A sample visual display is shown in Fig. 1.

### Experimental Design

The 60 native Cantonese listeners were divided randomly into two testing groups: one group participated in the word-viewing task and another group in the picture-viewing task. The thirty participants in each group were again assigned randomly to three groups of ten. Each group randomly received an equal number of sentences for each context condition in the 3 (Context type) × 4 (Probe type) mixed factorial design. The order of presentation of sentences and the positions of different types of visual probes were counterbalanced across all participants. No participant heard the same homophone or saw the same set of visual probes twice. Each type of visual probe had an equal chance to appear in each of the four positions on the computer screen.

### Apparatus

All experimental sentences were read by a native female Cantonese speaker at her normal conversation pace and recorded on a voice recorder. The spoken sentences were transformed and digitized into a computer. A Philips 220S (22-inch wide LCD monitor) was used to display the stimuli. All stimuli were black on a white background. The word probes were shown on the computer screen in the size of 8 cm × 8 cm with a 6 cm white space between any probes that were side by side. The picture probes were simple 12 cm × 12 cm line drawings with a 4 cm white space. A computer program was constructed by Experiment Builder to control the presentation of the stimuli. The eye-tracking machine (Eye-Link 1000 system, SR Research), with 1000 Hz temporal resolution, was connected to the computer to record the participants’ eye movements. The computer monitor was positioned approximately 60 cm away from the participants’ eyes.

### Procedure

The participants completed the tasks individually in a quiet room in the psychology laboratory. The experimenter explained the procedure in Cantonese to each participant and conducted a 9-point calibration test on the eye tracker before the experiment. The participants were told that they had no specific tasks to perform in any trial but were simply required to listen carefully to the sentences while looking at the words or pictures on the screen. In addition, they were told that they were free to look anywhere on the computer screen, but they must not close their eyes or take their eyes off the screen throughout the experiment.

The procedure of the experiment was as follows: first, at the beginning of each trial, a fixation cross appeared on the computer screen for 500 milliseconds to capture the participant’s attention, followed by a 500 millisecond blank screen. Then, a sentence was presented aurally from a speaker, and at the same time a set of 4 Cantonese words or 4 line-drawing pictures (visual probe) was presented on the 4 quadrants on the computer screen for 7 seconds, this being 2 seconds longer than the duration of the auditory sentence. A fixation point appeared on the center of the computer screen before every trial, allowing for recalibration when necessary. The whole experiment lasted about 10 minutes.

### Data coding and analysis

The eye-tracking machine recorded different eye-movement parameters from each participant’s left eye; for example, fixation points, saccades and fixations time, using the algorithm provided by the Eyelink 1000 machine. Following the conventional coding method used in this type of research^28,33,39^, each eye fixation was denoted by a dot and they were then linked as a time line representing the order of fixation occurrences. All the eye fixations, starting from onset of the spoken sentence to the end, were recorded. However, to examine on how the context affected homophone processing more closely, we magnified the time window ranging from 60 ms before the onset of the homophone to the offset of the homophone by a time line for data analysis because it allows us to observe for any shift in overt attention to each different type of visual probes across the critical timeframe of the onset of the homophone. This time line was plotted against the exact timeframe of the target word (Cantonese homophone), showing the dynamic changes of the eye-fixations. Eye-fixations located at the cell of the grid of visual probes (line-drawing pictures and Cantonese words) were coded as fixations to those specific visual probes.

## Results

### Data for the word-viewing version

**Figure 2:**
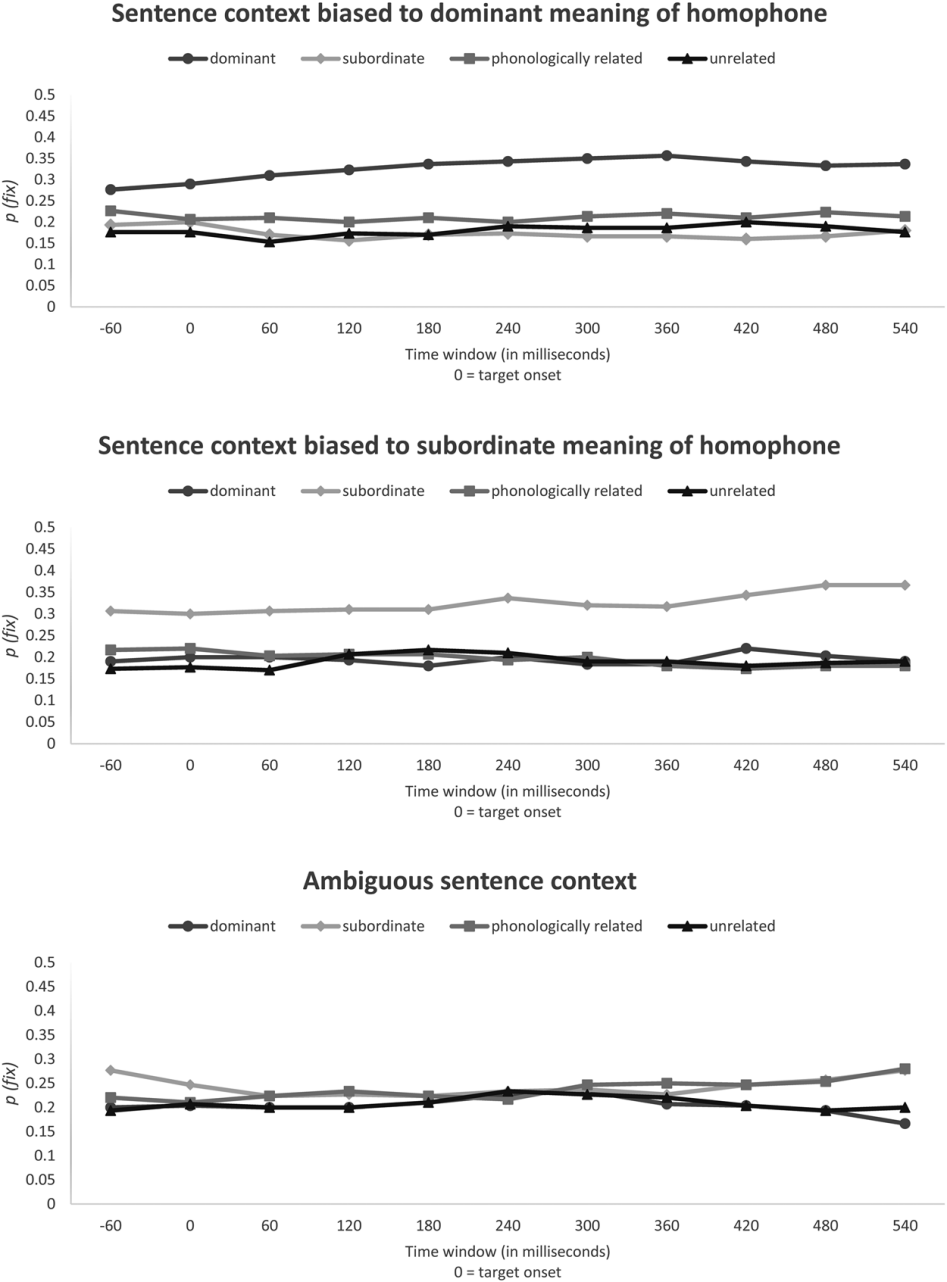
Time-course graphs illustrating the eye-fixation probabilities as a function of context type and probe type (word-viewing version).

Three time-course graphs are presented in Fig. 2. These graphs show the fixation proportions at 60 ms intervals for different types of visual probe (Cantonese words) relative to the pre-onset, onset and offset time of the target homophone under different context types. *P(fix)* is the probability that participants fixate on a specific type of visual probe (Cantonese word).

For the statistical analyses, we adopted the similar approach used in previous studies on this topic to make a clear comparison among relevant studies^28,29,39^. Hence. we computed the mean proportion of fixations for each type of word probes under three types of sentence context over a time interval starting from 60 ms before the target homophone onset to 60 ms after, in order to show the overall context effects from the time points before (and after) the occurrence of the target homophone. We split this time interval into eleven 60 ms time windows and conducted two-way ANOVA (using R) to test the interactions between context type and probe type for each time window. Table 1 shows the interaction effects between context type and probe type for each and every time window for word probe version of the experiment.

**Table 1:** Interactions between context type and probe type for each time window for word-viewing version.

Time window (relative to target onset)	Interaction	
−60~0 ms	F_1_ (6, 174) = 2.41, p = 0.029	F_2_ (6, 174) = 2.43, p = 0.028
0~60 ms	F_1_ (6, 174) = 2.36, p = 0.032	F_2_ (6, 174) = 2.33, p = 0.035
60~120 ms	F_1_ (6, 174) = 3.85, p = 0.001	F_2_ (6, 174) = 3.85, p = 0.001
120~180 ms	F_1_ (6, 174) = 4.96, p < 0.001	F_2_ (6, 174) = 4.57, p < 0.001
180~240 ms	F_1_ (6, 174) = 5.77, p < 0.001	F_2_ (6, 174) = 4.61, p < 0.001
240~300 ms	F_1_ (6, 174) = 6.53, p < 0.001	F_2_ (6, 174) = 4.94, p < 0.001
300~360 ms	F_1_ (6, 174) = 7.59, p < 0.001	F_2_ (6, 174) = 5.46, p < 0.001
360~420 ms	F_1_ (6, 174) = 8.27, p < 0.001	F_2_ (6, 174) = 6.08, p < 0.001
420~480 ms	F_1_ (6, 174) = 7.97, p < 0.001	F_2_ (6, 174) = 6.23, p < 0.001
480~540 ms	F_1_ (6, 174) = 7.96, p < 0.001	F_2_ (6, 174) = 7.55, p < 0.001
540~600 ms	F_1_ (6, 174) = 8.87, p < 0.001	F_2_ (6, 174) = 8.15, p < 0.001

The interaction effects between sentence context type and probe type are significant for all time windows, so we further look at the simple main effects of probe type for each type of sentence context (with Bonferroni’s correction) for each time window to test whether the proportion of fixation was significantly higher for certain types of visual probes than for others^40^. Table 2 shows the analyses for sentence contexts biased toward dominant meanings of homophones with different word probes. The *F*-test suggested significant differences for all 11 time-windows. The results of post hoc tests showed that the proportion of fixations on word probes related semantically to the dominant meaning of the target homophone was significantly higher than for the other three types of word probes (*ps* < 0.016) 120 ms after target onset.

**Table 2:** Time-window analyses for sentence context biased toward dominant meaning of homophones with different word probes.

Time window (relative to target onset)	Means				ANOVA	
	Semantically related to dominant meaning	Semantically related to subordinate meaning	Phonologically similar to target homophone	Unrelated to target homophones		
−60~0 ms	0.28	0.19	0.23	0.18	F_1_(3, 116) = 3.4 p = 0.02	F_2_(3, 116) = 2.4 p = 0.069
0~60 ms	0.29	0.20	0.21	0.18	F_1_(3, 116) = 3.9 p = 0.01	F_2_(3, 116) = 3.2 p = 0.027
60~120 ms	0.31	0.17	0.21	0.15	F_1_(3, 116) = 7.8 p < 0.001	F_2_(3, 116) = 7.0 p < 0.001
120~180 ms	0.32	0.16	0.20	0.17	F_1_(3, 116) = 9.2 p < 0.001	F_2_(3, 116) = 9.0 p < 0.001
180~240 ms	0.34	0.17	0.21	0.17	F_1_(3, 116) = 10.1 p < 0.001	F_2_(3, 116) = 10.7 p < 0.001
240~300 ms	0.34	0.17	0.20	0.19	F_1_(3, 116) = 11.2 p < 0.001	F_2_(3, 116) = 11.5 p < 0.001
300~360 ms	0.35	0.17	0.21	0.19	F_1_(3, 116) = 12.7 p < 0.001	F_2_(3, 116) = 14.1 p < 0.001
360~420 ms	0.36	0.17	0.22	0.19	F_1_(3, 116) = 10.8 p < 0.001	F_2_(3, 116) = 15.5 p < 0.001
420~480 ms	0.34	0.16	0.21	0.20	F_1_(3, 116) = 10.0 p < 0.001	F_2_(3, 116) = 13.4 p < 0.001
480~540 ms	0.33	0.17	0.22	0.19	F_1_(3, 116) = 8.0 p < 0.001	F_2_(3, 116) = 11.6 p < 0.001
540~600 ms	0.34	0.18	0.21	0.18	F_1_(3, 116) = 8.8 p < 0.001	F_2_(3, 116) = 10.9 p < 0.001

Table 3 shows the analyses for sentence context biased toward subordinate meanings of Cantonese homophones with word probes. Again, an *F*-test indicated significant differences for all time windows. The post hoc tests implied that the probability of fixations on word probes related semantically to the subordinate meaning of the target homophone was significantly higher than for the other three types of word probes (*ps* < 0.016) for the last 7 time windows.

**Table 3:** Time-window analyses for sentence context biased toward subordinate meaning of homophones with different word probes.

Time window (relative to target onset)	Means				ANOVA	
	Semantically related to dominant meaning	Semantically related to subordinate meaning	Phonologically similar to target homophone	Unrelated to target homophones		
−60~0 ms	0.19	0.31	0.22	0.17	F_1_ (3, 116) = 4.6 p = 0.004	F_2_ (3, 116) = 5.6 p = 0.001
0~60 ms	0.20	0.30	0.22	0.18	F_1_ (3, 116) = 4.0 p = 0.009	F_2_ (3, 116) = 4.1 p = 0.008
60~120 ms	0.20	0.31	0.20	0.17	F_1_ (3, 116) = 4.7 p = 0.004	F_2_ (3, 116) = 4.4 p = 0.006
120~180 ms	0.19	0.31	0.21	0.21	F_1_ (3, 116) = 4.0 P = 0.009	F_2_ (3, 116) = 3.7 p = 0.013
180~240 ms	0.18	0.31	0.21	0.22	F_1_ (3, 116) = 3.6 P = 0.015	F_2_ (3, 116) = 4.5 p = 0.005
240~300 ms	0.20	0.34	0.19	0.21	F_1_ (3, 116) = 5.4 P = 0.002	F_2_ (3, 116) = 6.1 p = 0.001
300~360 ms	0.18	0.32	0.20	0.19	F_1_ (3, 116) = 4.6 P = 0.005	F_2_ (3, 116) = 7.9 p < 0.001
360~420 ms	0.18	0.32	0.18	0.19	F_1_ (3, 116) = 5.4 P = 0.002	F_2_ (3, 116) = 7.9 p < 0.001
420~480 ms	0.22	0.34	0.17	0.18	F_1_ (3, 116) = 7.7 P < 0.001	F_2_ (3, 116) = 10.3 p < 0.001
480~540 ms	0.20	0.37	0.18	0.19	F_1_ (3, 116) = 10.9 P < 0.001	F_2_ (3, 116) = 11.5 p < 0.001
540~600 ms	0.19	0.37	0.18	0.19	F_1_ (3, 116) = 11.3 P < 0.001	F_2_ (3, 116) = 13.4 p < 0.001

Table 4 shows the analyses for sentences with ambiguous context. No significant differences were found except for the first and last time windows. Post hoc tests suggested that during 540–600 ms, the probability of fixations for probes had a significantly lower semantic relationship to the dominant meaning of the target than those for word probes phonologically related to the target (*p* < 0.016).

**Table 4:** Time-window analyses for ambiguous sentence context with different word probes.

Time window (relative to target onset)	Means				ANOVA	
	Semantically related to dominant meaning	Semantically related to subordinate meaning	Phonologically similar to target homophone	Unrelated to target homophones		
−60~0 ms	0.20	0.28	0.22	0.19	F_1_ (3, 116) = 3.0 p = 0.035	F_2_ (3, 116) = 1.9 p = 0.131
0~60 ms	0.20	0.25	0.21	0.21	F_1_ (3, 116) = 0.9 p = 0.448	F_2_ (3, 116) = 0.6 p = 0.636
60~120 ms	0.20	0.22	0.22	0.20	F_1_ (3, 116) = 0.4 p = 0.758	F_2_ (3, 116) = 0.3 p = 0.843
120~180 ms	0.20	0.23	0.23	0.20	F_1_ (3, 116) = 0.6 p = 0.629	F_2_ (3, 116) = 0.5 p = 0.696
180~240 ms	0.21	0.22	0.22	0.21	F_1_ (3, 116) = 0.1 p = 0.956	F_2_ (3, 116) = 0.1 p = 0.963
240~300 ms	0.23	0.23	0.22	0.23	F_1_ (3, 116) = 0.1 p = 0.957	F_2_ (3, 116) = 0.1 p = 0.955
300~360 ms	0.23	0.24	0.25	0.23	F_1_ (3, 116) = 0.1 p = 0.951	F_2_ (3, 116) = 0.1 p = 0.943
360~420 ms	0.21	0.23	0.25	0.22	F_1_ (3, 116) = 0.6 p = 0.649	F_2_ (3, 116) = 0.6 p = 0.631
420~480 ms	0.20	0.25	0.25	0.20	F_1_ (3, 116) = 1.0 p = 0.402	F_2_ (3, 116) = 1.1 p = 0.363
480~540 ms	0.19	0.26	0.25	0.19	F_1_ (3, 116) = 2.0 p = 0.120	F_2_ (3, 116) = 2.1 p = 0.107
540~600 ms	0.17	0.28	0.28	0.20	F_1_ (3, 116) = 5.4 p = 0.002	F_2_ (3, 116) = 4.6 p = 0.005

### Data for the picture-viewing version

**Figure 3:**
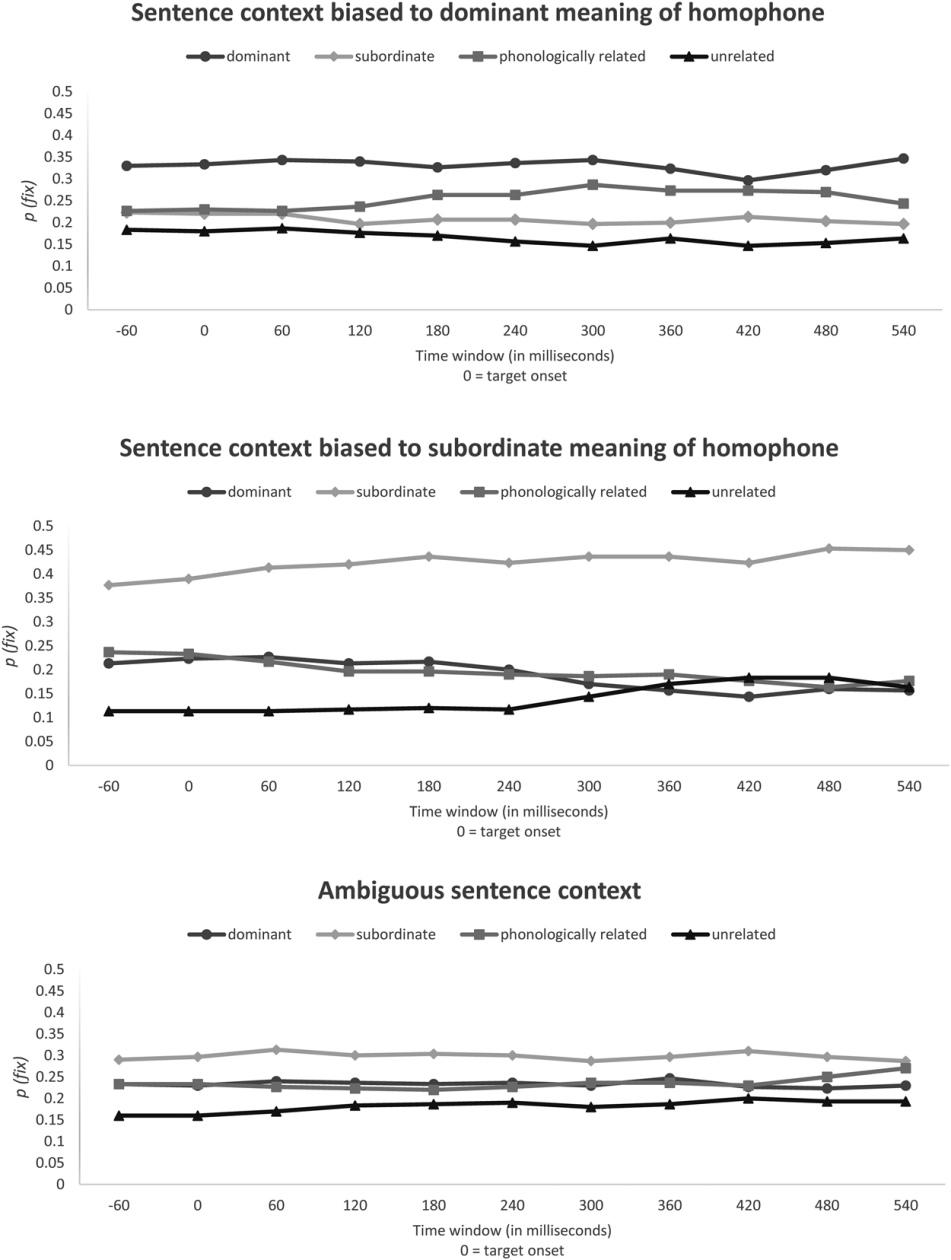
Time-course graphs illustrating the eye-fixation probabilities as a function of context type and probe type (picture-viewing version).

Three time-course graphs are presented in Fig. 3. These graphs show the fixation proportions at 60 ms intervals for the different types of visual probes (line-drawing pictures) relative to the pre-onset, onset and offset times of the target homophone under different context types. *P(fix)* denotes the probability that the eye fixation is on a specific type of visual probes in each time window.

Again, we computed the mean proportion of fixations for each type of picture probes under the three conditions of sentence context over the region of interest (i.e. the time interval starting from 60 ms before the target homophone onset to 60 ms after the end of the homophone). So, we split this time interval into thirteen 60 ms time windows and conducted two-way ANOVA tests for each time window to examine the interaction effects between context type and probe type. Table 5 shows the interaction effects for each and every time window for picture word probe version of this experiment.

**Table 5:** Interactions between context type and probe type for each time window for picture-viewing version.

Time window (relative to target onset)	Interaction	
−60~0 ms	F_1_(6, 174) = 3.28, p = 0.004	F_2_(6, 174) = 4.43, p < 0.001
0~60 ms	F_1_(6,174) = 3.62, p = 0.002	F_2_(6, 174) = 4.54, p < 0.001
60~120 ms	F_1_(6, 174) = 4.81, p < 0.001	F_2_(6, 174) = 5.99, p < 0.001
120~180 ms	F_1_(6, 174) = 5.86, p < 0.001	F_2_(6, 174) = 7.47, p < 0.001
180~240 ms	F_1_(6, 174) = 6.64, p < 0.001	F_2_(6, 174) = 7.12, p < 0.001
240~300 ms	F_1_(6, 174) = 6.25, p < 0.001	F_2_(6, 174) = 7.29, p < 0.001
300~360 ms	F_1_(6, 174) = 8.29, p < 0.001	F_2_(6, 174) = 10.76, p < 0.001
360~420 ms	F_1_(6, 174) = 6.73, p < 0.001	F_2_(6, 174) = 9.17, p < 0.001
420~480 ms	F_1_(6, 174) = 5.89, p < 0.001	F_2_(6, 174) = 7.81, p < 0.001
480~540 ms	F_1_(6, 174) = 7.76, p < 0.001	F_2_(6, 174) = 9.90, p < 0.001
540~600 ms	F_1_(6, 174) = 8.40, p < 0.001	F_2_(6, 174) = 10.39, p < 0.001

Since the interaction effects between context type and probe type for each and every time window for picture-viewing version of the experiment are significant, we further look at the simple main effects of probe type for each type of sentence context. Table 6 shows analyses for sentence context biased toward the dominant meanings of homophones with the picture versions of the visual probes. *F*-test results indicated a significant difference for all 11 time-windows. The results of post hoc tests (with Bonferroni’s correction) suggested that, for most of the time windows, the proportion of fixations for picture probes related semantically to the dominant meaning of the target homophone was significantly higher than those for probes related to subordinated meaning or unrelated probes (*ps* < 0.016). However, no significant difference was observed between probes related semantically to the dominant meaning of homophones and those related phonologically to the target.

**Table 6:** Time-window analyses for sentence context biased toward dominant meaning of homophones with different picture probes.

Time window (relative to target onset)	Means				ANOVA	
	Semantically related to dominant meaning	Semantically related to subordinate meaning	Phonologically similar to target homophone	Unrelated to target homophones		
−60~0 ms	0.33	0.22	0.23	0.18	F_1_ (3, 116) = 5.2 p = 0.002	F_2_ (3, 116) = 3.6 p = 0.015
0~60 ms	0.33	0.22	0.23	0.18	F_1_ (3, 116) = 5.9 p = 0.001	F_2_ (3, 116) = 3.8 p = 0.012
60~120 ms	0.34	0.22	0.23	0.19	F_1_ (3, 116) = 7.2 p < 0.001	F_2_ (3, 116) = 4.7 p = 0.004
120~180 ms	0.34	0.20	0.24	0.18	F_1_ (3, 116) = 7.7 P < 0.001	F_2_ (3, 116) = 5.1 p = 0.002
180~240 ms	0.33	0.21	0.26	0.17	F_1_ (3, 116) = 6.7 P < 0.001	F_2_ (3, 116) = 4.8 p = 0.003
240~300 ms	0.34	0.21	0.26	0.16	F_1_ (3, 116) = 8.8 P < 0.001	F_2_ (3, 116) = 6.2 p = 0.001
300~360 ms	0.34	0.20	0.29	0.15	F_1_ (3, 116) = 11.3 P < 0.001	F_2_ (3, 116) = 9.3 p < 0.001
360~420 ms	0.32	0.20	0.27	0.16	F_1_ (3, 116) = 8.0 P < 0.001	F_2_ (3, 116) = 6.2 p = 0.001
420~480 ms	0.30	0.21	0.27	0.15	F_1_ (3, 116) = 7.8 P < 0.001	F_2_ (3, 116) = 5.5 p = 0.001
480~540 ms	0.32	0.20	0.27	0.15	F_1_ (3, 116) = 9.2 P < 0.001	F_2_ (3, 116) = 6.2 p = 0.001
540~600 ms	0.35	0.20	0.24	0.16	F_1_ (3, 116) = 10.3 P < 0.001	F_2_ (3, 116) = 6.5 p < 0.001

Again, Table 7 shows the analyses for the sentence context biased toward the subordinate meaning of homophones with picture probes. Again, significant differences were found for all time windows. The post hoc tests implied that the probability of fixations for picture probes related semantically to subordinate meaning of the target homophone was significantly higher than those for the other three types of picture probe (*ps* < 0.016) in all time windows.

**Table 7:** Time-window analyses for sentence context biased toward subordinate meaning of homophones with different picture probes.

Time window (relative to target onset)	Means				ANOVA	
	Semantically related to dominant meaning	Semantically related to subordinate meaning	Phonologically similar to target homophone	Unrelated to target homophones		
−60~0 ms	0.21	0.38	0.24	0.11	F_1_ (3, 116) = 20.2 p < 0.001	F_2_ (3, 116) = 13.1 p < 0.001
0~60 ms	0.22	0.39	0.23	0.11	F_1_ (3, 116) = 21.9 p < 0.001	F_2_ (3, 116) = 15.1 p < 0.001
60~120 ms	0.23	0.41	0.22	0.11	F_1_ (3, 116) = 27.8 p < 0.001	F_2_ (3, 116) = 16.9 p < 0.001
120~180 ms	0.21	0.42	0.20	0.12	F_1_ (3, 116) = 29.2 P < 0.001	F_2_ (3, 116) = 18.9 p < 0.001
180~240 ms	0.22	0.44	0.20	0.12	F_1_ (3, 116) = 36.0 P < 0.001	F_2_ (3, 116) = 21.5 p < 0.001
240~300 ms	0.20	0.42	0.19	0.12	F_1_ (3, 116) = 34.3 P < 0.001	F_2_ (3, 116) = 20.6 p < 0.001
300~360 ms	0.17	0.44	0.19	0.14	F_1_ (3, 116) = 33.2 P < 0.001	F_2_ (3, 116) = 20.5 p < 0.001
360~420 ms	0.16	0.44	0.19	0.17	F_1_ (3, 116) = 25.9 P < 0.001	F_2_ (3, 116) = 17.1 p < 0.001
420~480 ms	0.14	0.42	0.18	0.18	F_1_ (3, 116) = 22.8 P < 0.001	F_2_ (3, 116) = 15.1 p < 0.001
480~540 ms	0.16	0.45	0.16	0.18	F_1_ (3, 116) = 26.0 P < 0.001	F_2_ (3, 116) = 19.0 p < 0.001
540~600 ms	0.16	0.45	0.18	0.16	F_1_ (3, 116) = 27.5 P < 0.001	F_2_ (3, 116) = 19.9 p < 0.001

Finally, the analyses for conditions with ambiguous sentential context is shown in Table 8. *F*-test results indicated a significant difference between types of probes for all time windows (*ps* < 0.05). Post hoc tests suggested that the fixation probability for unrelated probes was significantly lower than that for the picture probes related semantically to the subordinate meaning of the target (*ps* < 0.016).

**Table 8:** Time-window analyses for ambiguous sentence context with different picture probes.

Time window (relative to target onset)	Means				ANOVA	
	Semantically related to dominant meaning	Semantically related to subordinate meaning	Phonologically similar to target homophone	Unrelated to target homophones		
−60~0 ms	0.23	0.29	0.23	0.16	F_1_ (3, 116) = 4.4 p = 0.006	F_2_ (3, 116) = 3.3 p = 0.022
0~60 ms	0.23	0.30	0.23	0.16	F_1_ (3, 116) = 4.5 p = 0.005	F_2_ (3, 116) = 3.7 p = 0.014
60~120 ms	0.24	0.31	0.23	0.17	F_1_ (3, 116) = 6.0 p = 0.001	F_2_ (3, 116) = 4.3 p = 0.007
120~180 ms	0.24	0.30	0.22	0.18	F_1_ (3, 116) = 4.2 p = 0.007	F_2_ (3, 116) = 2.6 p = 0.054
180~240 ms	0.23	0.30	0.22	0.19	F_1_ (3, 116) = 3.4 p = 0.021	F_2_ (3, 116) = 2.9 p = 0.037
240~300 ms	0.24	0.30	0.23	0.19	F_1_ (3, 116) = 2.9 p = 0.039	F_2_ (3, 116) = 2.4 p = 0.071
300~360 ms	0.23	0.29	0.24	0.18	F_1_ (3, 116) = 3.3 p = 0.023	F_2_ (3, 116) = 2.3 p = 0.081
360~420 ms	0.25	0.30	0.24	0.19	F_1_ (3, 116) = 3.1 p = 0.030	F_2_ (3, 116) = 2.5 p = 0.067
420~480 ms	0.23	0.31	0.23	0.20	F_1_ (3, 116) = 3.4 p = 0.021	F_2_ (3, 116) = 2.9 p = 0.036
480~540 ms	0.22	0.30	0.25	0.19	F_1_ (3, 116) = 2.8 p = 0.045	F_2_ (3, 116) = 2.4 p = 0.069
540~600 ms	0.23	0.29	0.27	0.19	F_1_ (3, 116) = 2.8 p = 0.042	F_2_ (3, 116) = 2.1 p = 0.106

## General Discussion

This study further investigated the time course of context effects and spoken word recognition during Chinese sentence comprehension. We used Cantonese monosyllabic homophones (which is different from a related study of Yip and Zhai^33^ in which they used disyllabic Mandarin homophones as stimuli) as the testing item because monosyllabic homophones in Cantonese are much more ambiguous at a lexical-morphemic level. The main goal of the study was to use a different research paradigm and a different language in order to get converging evidence about this fundamental and important research question on the study of language processing. Hence, we used Cantonese homophones (a typical class of ambiguous word) in this study to examine lexical ambiguity resolution due to the pervasive homophony phenomenon in this language. Moreover, we employed the visual world paradigm to explore this issue from a different dimension, i.e. to measure participants’ eye fixation on visual stimuli when they were listening concurrently to sentences ending with Cantonese homophones.

Consistent and convergent results from both eye-tracking tasks (word-viewing and picture-viewing versions) clearly showed a main effect for context types and probe types as well as an interaction effect between the two variables. In both the word-viewing and picture-viewing versions, the probabilities of eye fixations clearly reflected that the sentence context facilitated the lexical access to Cantonese homophones at a very early stage. These findings also demonstrated a strong and solid context effect to pre-select the contextually-appropriate meaning of the ambiguous word even before the occurrence of the homophone (i.e. −60 ms), which is consistent with the context-dependency hypothesis. Furthermore, the results did not show any reliable phonological effect on the processing of Chinese homophones if the semantic context was strong enough to pre-select the correct meaning of the ambiguous word. This has been the first eye-tracking study on this topic using Cantonese homophones as the challenging case. Combined with the results of other relevant studies using different paradigms (e.g. behavioral measures), different experimental designs^41^ and different major Chinese dialects^33^, the evidence adds new cross-language evidence to the literature on lexical ambiguity resolution in Chinese, a Sino-Tibetan language. Together with all relevant research studies in Chinese homophone processing (including Cantonese and Mandarin)^19^, we conclude that native Chinese listeners are sensitive early on to contextually biased meaning, probably within the acoustic boundary of the target ambiguous word (or even before its occurrence). Altogether, the present series of evidences (including behavioral measures and eye movement measure) obviously show that preceding context limits the degree of initial activations among various meanings (or semantic representations) associated with the same ambiguous word (homophone). It clearly demonstrate that contextual information penetrates lexical access and exerts simultaneous influences on the lexical selection interactively^16,17^.

In conclusion, the eye movement data from the present study offer clear support to the view that native Cantonese listeners will never access all the related meanings of a homophone exhaustively when they hear the word in a contextually constraining sentence. On the contrary, when they hear a Chinese homophone, with multiple-meanings, they will automatically and rapidly use the semantic information provided by the sentence context to select or pre-select a contextually appropriate meaning for the ongoing spoken language comprehension. This interactive-activation processing approach can assist our language processors to disambiguate various homophone meanings effectively and efficiently during Chinese sentence processing^24,33,41^. Based on all the existing findings obtained from different paradigms, it is of much use to construct a model to explain the lexical ambiguity resolution as well as spoken language processing in Chinese (cf. TRACE^17^, and Shortlist model^42^).

In the future, we plan to design and conduct some laboratory experiments to investigate the lexical ambiguity issue further by researching another type of ambiguous words: Chinese homographs. This is because most of the western literature has focused on homographs in this line of research^11^, and the study of disyllabic homophones in Cantonese can give further information about the effects of the compounding nature of the Chinese language. Consequently, a more comprehensive picture of spoken language processing can be plotted solidly from a cross-linguistic perspective.

## Electronic supplementary material

Appendix
