# Supplementary material for: Processing Homophones Interactively: Evidence from eye-movement data
Source: Sci Rep. 2018 Jun 28;8:9812. doi: 10.1038/s41598-018-27768-5 (PMC6023861; doi:10.1038/s41598-018-27768-5)
Supplement: Supplementary file 1 — Appendix [file 41598_2018_27768_MOESM1_ESM.doc]

Processing Homophones Interactively:

Evidence from eye-movement data

Michael C. W. Yip

Department of Psychology, The Education University of Hong Kong

and

Mingjun ZHAI

Department of Psychology, Rice University

Appendix: Spoken Chinese Homophones used in the Experiment

baan1 baan2

bo3 boh1

cheung1 cheung4

chi4 do2

foh3 gau2

jeung3 jeung6

kei4 kwan4

ma5 min6

mo6 ping4 saam1 sau2

seung1 si1

sin3 sui3

tong4 wa6

wan4 woh1

woo4 yuen4

*Note: The number of each syllable represents the lexical tone
